# Insights in the underlying pathophysiology of brain malformations associated with *VRK1*-related syndrome derived from fetal neuropathology

**DOI:** 10.1093/jnen/nlaf014

**Published:** 2025-03-06

**Authors:** Aude Tessier, Olivier Monestier, François Guillaume Debray, Fréderic Vauthier, Valerie Benoit, Kim Van Berkel, Leila Ghassemi, Stefanie Brock

**Affiliations:** Institut de Pathologie et de Génétique, Gosselies, Belgium; Institut de Pathologie et de Génétique, Gosselies, Belgium; Metabolic Unit, Department of Medical Genetics, Sart-Tilman University Hospital, Liège, Belgium; Institut de Pathologie et de Génétique, Gosselies, Belgium; Institut de Pathologie et de Génétique, Gosselies, Belgium; Clinical Sciences, Research Group Genetics, Reproduction and Development, Centre for Medical Genetics, Vrije Universiteit Brussel (VUB), Universitair Ziekenhuis Brussel (UZ Brussel), Brussels, Belgium; Department of Gynecology and Obstetrics, , Hôpital Citadelle, Liège, Belgium; Department of Pathology, Universitair Ziekenhuis Brussel (UZ Brussel), Brussels, Belgium; Experimental Pathology (EXPA), Vrije Universiteit Brussel (VUB), Brussels, Belgium

To the Editor:

*VRK1* encodes a serine/threonine kinase that is ubiquitously expressed in all human cells. VRK1 is involved in cellular proliferation, DNA replication and damage response, chromatin remodeling, and Cajal body assembly and maintenance.^1^

Biallelic variants in *VRK1* have been described in patients with heterogeneous phenotypes with childhood- or adult-onset progressive upper and lower motor neuron disease, including spinal muscular atrophy, amyotrophic lateral sclerosis, and hereditary spastic paraplegia.^1^ Additionally, neurodevelopmental disorders, including rare cases of microcephaly, pontocerebellar hypoplasia, and simplified gyral pattern, have been reported.^2–5^

To date, only 2 fetal cases have been described. They presented with prenatal onset of microcephaly and cortical dysplasia, and diencephalic–mesencephalic junction dysplasia, microcephaly, and delayed gyration, respectively.^3^^,^^4^ Fetal postmortem findings, including neuropathological features, have not been previously reported.

Here, we report the case of 2 sibling fetuses with a *VRK1* variant. The parents are first cousins of North African origin. The mother has short stature due to a pathogenic variant in *PLAG1*. She had 5 pregnancies: 1 boy with short stature, affected by the Silver-Russel-type 4 syndrome, like his mother, 2 terminations of pregnancy (TOP) for prenatally detected microcephaly, and 2 spontaneous abortions.

For fetus 1, ultrasound and MRI were performed at 22 weeks gestational age (WG). Both ultrasound and MRI found microcephaly with diffuse delayed gyration. The size of the hindbrain was also reduced but this was less pronounced than the forebrain. The ventricles were enlarged, and complete agenesis of the corpus callosum was observed. TOP was performed at 26 WG and 6 days, according to Belgian law. On postmortem examination, this female fetus showed normal growth parameters for weight, foot-, and crown-rump length. She had microcephaly with a head size compatible with 21 WG. Facial dysmorphisms included downsloping forehead, hypertelorism, and a flat nasal bridge. The thighs appeared thick. On internal examination, no visceral malformation was noted. Brain weight was reduced (40 g, normal for 19-20 WG).^6^ On neuropathological macroscopic examination, gyration was completely absent. The lateral, third, and fourth ventricles were not enlarged. On microscopic examination, the cortex was paucicellular. Examination was incomplete due to autolysis.

The following pregnancy was also complicated by an abnormal second-trimester ultrasound scan, which showed recurrence of microcephaly with delayed gyration. TOP was performed at 22 WG and 3 days. The male fetus had normal growth parameters, except for head circumference, which corresponded to 20 WG. He had some dysmorphic features, including a square-shaped face, a down-sloping forehead, a long and smooth philtrum, anteverted nostrils, and thin lips. He had an unusual morphological presentation with muscle hypertrophy, short neck, and joint retraction. No skeletal anomalies were observed on X-ray. Except for the brain, there were no discernible internal malformations at autopsy. Neuropathological examination of the brain of fetus 2 showed severe microencephaly (brain weight 21.5 g, normal for 17 WG) with completely absent gyration (Figure 1A). The size of the hindbrain was reduced (weight = 1.8 g; normal weight for 19 WG), and transversal length of the cerebellum was 20 mm (normal for 19 WG). On sagittal section, the corpus callosum could not be discerned. Microscopically, agenesis of the corpus callosum was confirmed with the presence of Probst bundles. The hippocampus was dysplastic, with irregular convolutions (Figure 1B). The basal ganglia and thalami were dysmorphic and the ganglionic eminences were compressed (Figure 1C). In addition, the capsula interna was fragmented (Figure 1D). The cortex was paucicellular and thin compared to an age-matched control (0.6-0.9 cm compared to 0.9-1.3 cm in the control, Figure 2A and D). No clear overmigration of neuroblasts into the leptomeninges was observed. Focally, the cortical surface was irregular, resembling polymicrogyria but without a clear undulating band of neurons or entrapment of pial vessels. At the infratentorial level, the pons was hypoplastic, but beginning myelination was present. Longitudinal and transverse fibers were reduced, and the pyramids were hypoplastic. Examination of the cerebellum was inconclusive due to severe artifacts. Immunohistochemical studies showed a low number of Calretinin (clone SP65)-positive interneurons in the cortex, and a reduced number of SATB2-positive neurons mainly located in layers 2 and 5/6 (Figure 2B and E). The radial glial network in the white matter was severely disrupted (Figure 2C and F). No abnormalities were seen at routine histological examination of the eyes and spinal cord. Examination of fetal muscle from both fetuses was complicated by severe artifacts but was suspicious for mild fiber diameter variability.

**Figure 1:**
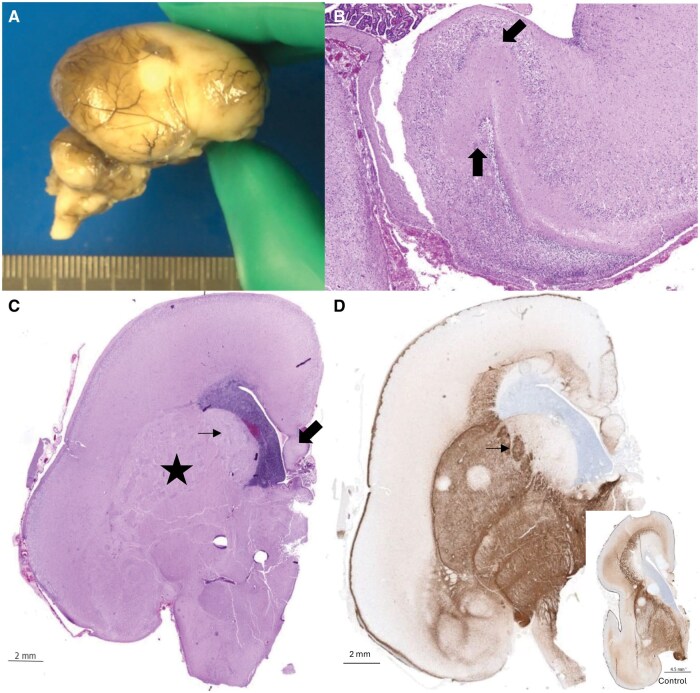
(A) Macroscopic examination of the brain of the second fetus at 22 GW shows severe microencephaly with a completely smooth cortical surface. (B) The hippocampus was dysmorphic (H&E, 2×). (C) The basal ganglia are globular (asterisk), compressing the ganglionic eminences (thin arrow). There was a complete agenesis of the corpus callosum with the presence of Probst bundles (thick arrow, H&E). (D) Neurofilament immunostaining (neurofilament 68K, clone 2F11) highlights the altered architecture of the tracts; and fragmentation of the internal capsule (arrow); compared to an age-matched control (inset).

**Figure 2:**
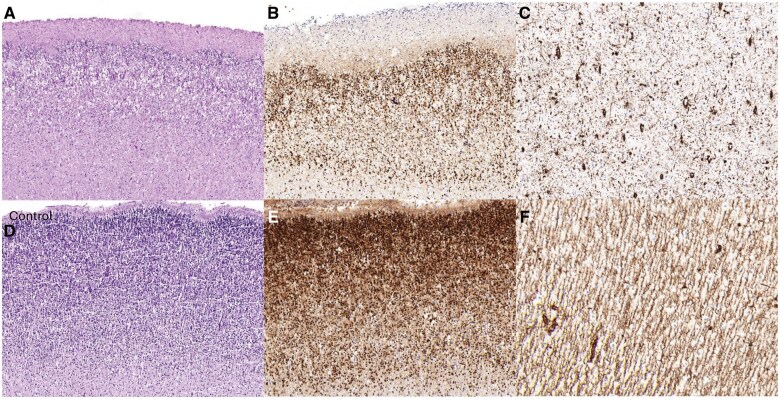
Histopathological findings in fetus 2 (A-C) and control (D-F). The cortical ribbon was thin with markedly reduced neuronal density (A) compared to the age-matched control (D), compatible with a simplified gyral pattern (H&E, 5×). There was a reduced number of SATB2 (Roche, clone EP281, 10×)-positive neurons (B), compared to a healthy age-matched control (E). The radial glial scaffold in the subcortical white matter was severely disrupted (C) compared to the regular linear pattern in an age-matched control (F, Vimentin, clone v9, 10×).

Both fetuses had normal molecular karyotypes. Quatuor whole exome sequencing identified a homozygous likely pathogenic (class IV) variant in *VRK1* (NM_003384.3, GRCh38):c.238C>G p.(Leu80Val) in both fetuses. Parents and older siblings were heterozygous carriers of this variant. Sequence alignment to the human reference genome, variant calling, and annotation were performed using an in-house bioinformatic pipeline. This variant is absent from control databases (gnomAD 4.1), involves a moderately conserved residue, and is predicted to be deleterious by most *in silico* tools (CADD score 24.6). The variant is located in a kinase domain. No other pathogenic variants were identified.

While the phenotype of our fetal cases corresponds to findings in previous reports, including microcephaly, delayed gyration, and pontocerebellar hypoplasia, we provide the first data on the histopathological features of these brain malformations. The pathophysiological mechanisms of how pathogenic variants in *VRK1* alter normal cortical and pontocerebellar development remain elusive. Neuronal progenitor proliferation has been reported to be impaired in a VRK1 knock-out zebrafish model, resulting in microcephaly and impaired motor function.^7^ Prior to this, neuronal migration and neurite outgrowth had been shown to be dependent on VRK1.^8^ Neuropathological examination in our cases supports these hypotheses derived from animal models and gives new insights into the underlying pathophysiological processes in humans. First, a reduced number of neurons, evocative of impaired neuronal proliferation, and second, completely absent gyration, suggestive of a neuronal migration deficit, are present in both fetal cases. Moreover, reduction of corticospinal tracts, disrupted internal capsule, and agenesis of the corpus callosum indicate abnormal axon guidance. In line with this, biallelic variants in *VRK1* have been shown to cause defective assembly of Cajal bodies in the nucleus, leading to alterations in axonal mRNA regulation and transport, possibly causing deficiencies in neurite outgrowth.^8–10^ In addition, variants in *VRK1* have also been suggested to disrupt downstream pathways implicated in longitudinal patterning of the brain axis.^3^ Although abnormal white matter tract development has been suggested to cause mildly delayed sulcation, our cases in this report showed a severe delay, unlikely to be exclusively secondary to abnormal cortical pinning.^11^

Both lissencephaly and simplified gyral pattern are characterized by a reduced number of gyri and sulci on imaging. Lissencephaly typically shows a thickened cortical ribbon due to abnormal neuronal migration. In the 2 fetal cases presented here, the cortical ribbon was rather thin and more evocative of a simplified gyral pattern.

Electromyography and muscle biopsies in patients with pathogenic variants in *VRK1* have been reported with features of neurogenic myopathy/atrophy^2^^,^^12^^,^^13^ or spinal muscular atrophy.^5^ Muscle biopsies in both cases did not show signs of severe neurogenic atrophy but were limited to mild muscle fiber diameter variation. This finding might be due to the early gestational age as well as the poor quality of the frozen muscle biopsy tissue. Nevertheless, we did not observe severe group atrophy, consistent with spinal muscular atrophy or amyotrophic lateral sclerosis as observed clinically in other patients. However, fetus 2 showed a very unusual macroscopic appearance of muscle hypertrophy that could underlie neuromuscular disease without histological anomalies at this stage. As this disease was progressive in the previously reported patients, some of its features could also be missing at an early stage.

Morphogenesis of the face is a complex and highly coordinated process that occurs between the third and eighth WG. Corpus callosum anomalies are frequently associated with hypertelorism, as in Greig syndrome (#175700) or Opitz GBBB syndrome (#300000). In fact, orbital telorism in the general population appears to be influenced by genes associated with midline anomalies.^14^ Disruption of primary cilia function in cranial neural crest cells, giving rise to most of the craniofacial skeleton and connective tissue, results in inappropriate cell proliferation.^15^ How *VRK1* may influence facial embryology is still unclear. An indirect effect through regional modification secondary to brain malformation could also influence neural crest cell development.

VRK1 is ubiquitously expressed in all cells and has a potential impact on DNA replication, cell cycle progression, and DNA damage repair.^10^ Nevertheless, all phenotypes linked to *VRK1* pathogenic variants have been associated with neurological impairment, and the pathophysiological mechanism of this restriction to the nervous system remains poorly understood.

The variant identified here is localized in a previously described cluster near the ATP-binding site. There is no clear phenotype-genotype correlation within the different described clusters, and genetic background has been discussed as being involved in phenotypic heterogeneity. In the family described herein, no additional pathogenic variant has been identified in another gene that may be involved in the phenotype of the fetuses. The specific effect of the variant may also play a major role in phenotypic variability, as the effects on protein stability or histone phosphorylation are very different between amino acid changes, even for nearby modifications.^1^ Furthermore, the phenotype seems to be quite homogenous in this family.

In conclusion, we describe the first fetal cases and the first report of a complete neuropathological examination associated with a *VRK1* homozygous pathogenic variant thereby extending and increasing the precision of the phenotype. Accurate descriptions of the phenotypes of rare diseases may lead to a better understanding of underlying pathological mechanisms.

## References

[1] Lazo PA , Morejón-García P. VRK1 variants at the cross road of Cajal body neuropathogenic mechanisms in distal neuropathies and motor neuron diseases. Neurobiol Dis. 2023;183:106172. 37257665 10.1016/j.nbd.2023.106172

[2] Gonzaga-Jauregui C , Lotze T, Jamal L, et al Mutations in VRK1 associated with complex motor and sensory axonal neuropathy plus microcephaly. JAMA Neurol. 2013;70:1491-1498. 24126608 10.1001/jamaneurol.2013.4598 PMC4039291

[3] Lawrence AK , Whitehead MT, Kruszka P, et al Prenatal diagnosis of diencephalic-mesencephalic junction dysplasia: fetal magnetic resonance imaging phenotypes, genetic diagnoses, and outcomes. Prenat Diagn. 2021;41:778-790. 33522008 10.1002/pd.5909

[4] Reches A , Hiersch L, Simchoni S, et al Whole-exome sequencing in fetuses with central nervous system abnormalities. J Perinatol. 2018;38:1301-1308. 30108342 10.1038/s41372-018-0199-3

[5] Renbaum P , Kellerman E, Jaron R, et al Spinal muscular atrophy with pontocerebellar hypoplasia is caused by a mutation in the VRK1 gene. Am J Hum Genet. 2009;85:281-289. 19646678 10.1016/j.ajhg.2009.07.006 PMC2725266

[6] Guihard-Costa AM , Ménez F, Delezoide AL. Organ weights in human fetuses after formalin fixation: standards by gestational age and body weight. Pediatr Dev Pathol. 2002;5:559-578. 12399830 10.1007/s10024-002-0036-7

[7] Carrasco Apolinario ME , Umeda R, Teranishi H, et al Behavioral and neurological effects of Vrk1 deficiency in zebrafish. Biochem Biophys Res Commun. 2023;675:10-18. 37429068 10.1016/j.bbrc.2023.07.005

[8] Vinograd-Byk H , Sapir T, Cantarero L, et al The spinal muscular atrophy with pontocerebellar hypoplasia gene VRK1 regulates neuronal migration through an amyloid-β precursor protein-dependent mechanism. J Neurosci. 2015;35:936-942. 25609612 10.1523/JNEUROSCI.1998-14.2015 PMC6605533

[9] El-Bazzal L , Rihan K, Bernard-Marissal N, et al Loss of Cajal bodies in motor neurons from patients with novel mutations in VRK1. Hum Mol Genet. 2019;28:2378-2394. 31090908 10.1093/hmg/ddz060

[10] Vinograd-Byk H , Renbaum P, Levy-Lahad E. Vrk1 partial knockdown in mice results in reduced brain weight and mild motor dysfunction, and indicates neuronal VRK1 target pathways. Sci Rep. 2018;8:11265. 30050127 10.1038/s41598-018-29215-x PMC6062608

[11] Warren DJ , Connolly DJA, Griffiths PD. Assessment of sulcation of the fetal brain in cases of isolated agenesis of the corpus callosum using in utero MR imaging. AJNR Am J Neuroradiol. 2010;31:1085-1090. 20093312 10.3174/ajnr.A1982 PMC7963955

[12] Demaegd K , Brilstra EH, Hoogendijk JE, et al Distal spinal muscular atrophy featured by predominant calf muscle involvement in VRK1 associated disease—case series and review. Neuromuscul Disord. 2022;32:527-532. 35641352 10.1016/j.nmd.2022.04.007

[13] Nguyen TP , Biliciler S, Wiszniewski W, et al Expanding phenotype of VRK1 mutations in motor neuron disease. J Clin Neuromuscul Dis. 2015;17:69-71. 26583493 10.1097/CND.0000000000000096 PMC4829393

[14] Knol MJ , Pawlak MA, Lamballais S, et al Genetic architecture of orbital telorism. Hum Mol Genet. 2022;31:1531-1543. 34791242 10.1093/hmg/ddab334 PMC9071440

[15] Brugmann SA , Allen NC, James AW, et al A primary cilia-dependent etiology for midline facial disorders. Hum Mol Genet. 2010;19:1577-1592. 20106874 10.1093/hmg/ddq030 PMC2846163

